# A Universal Principle to Accurately Synthesize Atomically Dispersed Metal–N_4_ Sites for CO_2_ Electroreduction

**DOI:** 10.1007/s40820-020-00443-z

**Published:** 2020-05-09

**Authors:** Wanzhen Zheng, Feng Chen, Qi Zeng, Zhongjian Li, Bin Yang, Lecheng Lei, Qinghua Zhang, Feng He, Xilin Wu, Yang Hou

**Affiliations:** 1 Key Laboratory of Biomass Chemical Engineering of Ministry of Education, College of Chemical and Biological Engineering, Zhejiang University, Hangzhou, 310027 People’s Republic of China; 2 College of Geography and Environmental Science, Zhejiang Normal University, Jinhua, 321004 People’s Republic of China; 3 College of Environment, Zhejiang University of Technology, Hangzhou, 310014 People’s Republic of China; 4 Institute of Zhejiang University - Quzhou, Quzhou, 324000 People’s Republic of China; 5 Ningbo Research Institute, Zhejiang University, Ningbo, 315100 People’s Republic of China; 6 Zhejiang Provincial Key Laboratory of Advanced Chemical Engineering Manufacture Technology, College of Chemical and Biological Engineering, Zhejiang University, Hangzhou, 310027 People’s Republic of China

**Keywords:** Atomic dispersion, Pyrrole-type metal*–*N_4_ structure, Catalytic site, CO_2_ electroreduction, Zn–CO_2_ battery

## Abstract

Atomically dispersed metal–nitrogen sites-anchored carbon materials have been developed as effective catalysts for CO_2_ electroreduction (CO_2_ER), but they still suffer from the imprecisely control of type and coordination number of N atoms bonded with central metal. Herein, we develop a family of single metal atom bonded by N atoms anchored on carbons (SAs–M–N–C, M = Fe, Co, Ni, Cu) for CO_2_ER, which composed of accurate pyrrole-type M–N_4_ structures with isolated metal atom coordinated by four pyrrolic N atoms. Benefitting from atomically coordinated environment and specific selectivity of M–N_4_ centers, SAs–Ni–N–C exhibits superior CO_2_ER performance with onset potential of − 0.3 V, CO Faradaic efficiency (F.E.) of 98.5% at − 0.7 V, along with low Tafel slope of 115 mV dec^−1^ and superior stability of 50 h, exceeding all the previously reported M–N–C electrocatalysts for CO_2_-to-CO conversion. Experimental results manifest that the different intrinsic activities of M–N_4_ structures in SAs–M–N–C result in the corresponding sequence of Ni > Fe > Cu > Co for CO_2_ER performance. An integrated Zn–CO_2_ battery with Zn foil and SAs–Ni–N–C is constructed to simultaneously achieve CO_2_-to-CO conversion and electric energy output, which delivers a peak power density of 1.4 mW cm^−2^ and maximum CO F.E. of 93.3%.

**Electronic supplementary material:**

The online version of this article (10.1007/s40820-020-00443-z) contains supplementary material, which is available to authorized users.

## Highlights

- A family of SAs–M–N–C consisted of carbon nanosheets supported atomic sites of isolated metal atom coordinated with four pyrrolic N atoms was fabricated.
- The SAs–Ni–N–C exhibited superior electrochemical CO_2_ electroreduction (CO_2_ER) activity and selectivity.

**Electronic supplementary material:**

The online version of this article (10.1007/s40820-020-00443-z) contains supplementary material, which is available to authorized users.

## Graphical abstract

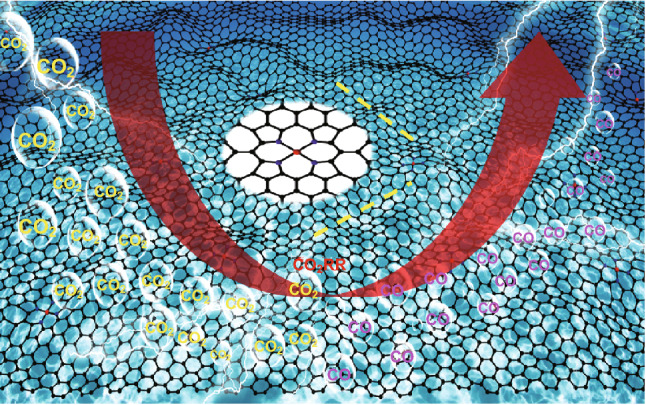

## Introduction

With the increasing concentration of atmospheric carbon dioxide (CO_2_), how to effectively reduce CO_2_ into available resources, such as carbon monoxide (CO) [1–4], formic acid (HCOOH) [5, 6], hydrocarbons (C_2_, C_3_) [7, 8], and alcohols (CH_3_OH, CH_3_CH_2_OH) [9, 10], by using electrochemical strategy has become a hot topic. Among all reported products from CO_2_ electroreduction (CO_2_ER), CO gas is a relatively easy product to be yielded due to the CO_2_-to-CO conversion only involved two-step procedure of proton-coupled electron transfer. Moreover, the produced gaseous CO can be handily separated and be further used as resource in other industrial applications, like Fischer–Tropsch process [11]. Originally, noble metals (Au, Ag, Pd, etc.)-based materials are widely used to catalyze CO_2_ into CO [12–14]; however, the application of these noble metal materials is highly hindered by their high cost and scarcity. Hence, significant efforts have been devoted to develop low-cost and highly effective alternative catalysts to replace the noble metals materials for CO_2_-to-CO conversion. Currently, atomically dispersed metal–nitrogen (M–N) sites-anchored carbon (M–N–C) materials is one of the most promising CO_2_ER electrocatalysts for CO production, owing to its simple synthetic procedure and excellent catalytic performance [15–19]. First, the M–N–C materials can be synthesized via a one-step pyrolysis of precursors contained carbon resources, nitrogen resources, and inorganic metal salt under optimized condition [20–24], and this strategy is a universal synthetic method that can be used to develop a series of M–N–C materials. Second, the electronic structure of central metal atom in M–N sites can be modified by the bonded N atoms, thus resulting in enhanced binding strength between the reaction intermediates and active M–N centers in their key step [25–28], promoting the catalytic activity and selectivity of M–N–C materials for CO_2_-to-CO conversion. Despite certain progress on developing M–N–C catalysts, it still suffers from imprecisely regulating the category and coordination number of ligating N atoms that bind to central metal atom. To be precise, several categories of N atoms such as pyridinic N, pyrrolic N, and graphitic N can provide coordinated possibility with metal atoms to form M–N structure during pyrolysis process; meanwhile, accurate ligand number between N and central metal atoms is difficult to be controlled.

Herein, we developed a universal approach to synthesize a series of single metal atom–N (SAs–M–N, M = Fe, Co, Ni, Cu) species immobilized on graphitized carbon supports (SAs–M–N–C) via an in situ pyrolysis of metalloporphyrin molecules and MCA polymer that was originated from the self-assemble of melamine (M) and cyanuric acid (CA). The SAs–M–N–C catalysts consisted of ultrathin carbon nanosheets supported accurate coordination structures of four pyrrole-type N atoms bonded with single metal atom (pyrrole-type M–N_4_). Benefitting from unique coordinated condition and discrepant intrinsic activity of pyrrole-type M–N_4_ sites in as-prepared SAs–M–N–C catalysts, SAs–Ni–N–C exhibited an excellent activity, selectivity, and stability for CO_2_-to-CO conversion, in which the conversion started at low potential of − 0.3 V along with a small Tafel slope of 115 mV dec^−1^; meanwhile, a high Faradaic efficiency (F.E.) of 98.5% for CO production and durable catalytic stability of 50 h were achieved at − 0.7 V. Experimental measurements revealed that the CO_2_ER performance ranking of SAs–M–N–C was corresponding to the sequence of Ni > Fe > Cu > Co owing to the intrinsic nature of pyrrole-type M–N_4_ structures, in which the high CO_2_ER performance catalyzed by SAs–Ni–N–C was appreciably superior to that of almost all previously reported M–N–C CO_2_ER electrocatalysts to date. The aberration-corrected high-angle annular dark field scanning transmission electron microscopy (AC HAADF-STEM) confirmed the atomic distribution of isolated metal atoms in SAs–M–N–C; X-ray photoelectron spectroscopy (XPS) and X-ray absorption spectroscopy (XAS) identified the accurate configuration of pyrrole-type M–N_4_ centers with individual metal atom bonded by four pyrrole-type N atoms. Furthermore, an integrated Zn–CO_2_ battery equipped with the cathode of SAs–Ni–N–C delivered a peak power density of 1.4 mW cm^−2^ and the maximum CO F.E. of 93.3% during its discharge process, realizing the practical feasibility of CO_2_ conversion and electric energy output.

## Experimental Section

### Materials

Deionized water was used for whole experiments. Melamine (C_3_H_6_N_6_) was purchased from Alfa Aesar Chemical Co., Ltd. Cyanuric acid (C_3_H_3_N_3_O_3_), iron(III) tetraphenylporphyrin chloride, cobalt(II) tetraphenylporphyrin, nickel(II) tetraphenylporphyrin, and copper(II) tetraphenylporphyrin were purchased from Tokyo Chemical Industry Co., Ltd. The above chemicals were directly used as received without any further purification.

### Preparation of SAs–M–N–C

The SAs–M–N–C samples were synthesized via a one-step in situ pyrolysis of metalloporphyrin molecules (Cu, Fe, Co, and Ni) on the surface of melamine (M) and cyanuric acid (CA)-polymerized polymer. First, 0.37 g (3.0 mmol) of M and 0.39 g (3.0 mmol) of CA were self-assembled in 40 mL of deionized water in an Erlenmeyer flask under ultrasonication condition to form a MCA polymer colloid. Then, the obtained MCA polymer colloid was separated by filtration and dried in vacuum at 60 °C for 10 h. Next, 1.0 g of solid MCA polymer was grinded with 0.2 g of metalloporphyrin to form a homogeneous mixed powder. Finally, the above mixture was placed in a tube furnace and heated at 700 °C for 2 h under N_2_ atmosphere with a heating rate of 5 °C min^−1^. After the calcination process, the corresponding black SAs-M-N-C product was obtained.

### Preparation of N–C

The N–C was synthesized by direct pyrolysis of MCA polymer without adding metalloporphyrin molecules under the same pyrolysis condition for SAs–M–N–C synthesis.

### Characterization

The field-emission scanning electron microscopy (FESEM) (SU-8010 Hitachi) and HRTEM (Tecnai G2 F20 S-TWIN) images were taken to identify the morphologies of samples. The X-ray diffraction (XRD) measurements were performed on ZETIUM DY 2186, 4 kW to display the crystal structures of samples. The metal content in samples was quantified by inductively coupled plasma atomic emission spectroscopy (ICP-AES) performed on Vista Axial. The XPS spectra of samples were collected on the Escalab 250Xi using an Al Kα radiation. The XAS results were obtained at the beamline 1W1B of the Q9 Beijing Synchrotron Radiation Facility (Beijing, China) using a transmission mode to detect the coordination environment of samples. Liquid-phase CO_2_ER products were identified by ^1^H NMR (600 MHz, Bruker AVANCE AV III 500), in which 600 µL of 0.5 M KHCO_3_ electrolyte after long-term CO_2_ER electrolysis was mixed with 70 µL of 10 mM dimethyl sulfoxide (DMSO) in D_2_O for ^1^H NMR analysis. The DMSO was used as an internal standard, and the solvent suppression was used to decrease the area of H_2_O peak to make the CO2ER products peaks more clearly. Notably, all of the liquid-phase CO_2_ER products can be identified by ^1^H NMR [29].

### Electrochemical Measurements

Electrochemical measurements were tested on CHI 760E electrochemical workstation with a three-electrode cell (counter electrode: Pt wire; reference electrode: Ag/AgCl; working electrode: 1 × 1 cm^2^ carbon paper loaded with catalyst). For the working electrode, homogeneous ink (10 mg mL^−1^) consisting of 10 mg of sample, 100 µL of 0.5% Nafion, and 900 µL of ethanol was prepared with sonication and stirring. Then, 100 µL of suspension solution was dropped onto the surface of carbon paper with loading amount of 1.0 mg cm^−2^. The polarization curves were measured in 0.5 M KHCO_3_ solution with a scan rate of 5 mV s^−1^. The ECSA-referred cyclic voltammetry (CV) curves were performed at the potential of − 0.35 V ~ − 0.45 V (vs. Ag/AgCl). The EIS spectra were measured with a frequency ranging from 100 kHz to 10 mHz and an AC voltage with 5 mV. The mentioned potentials versus reversible hydrogen electrode (RHE) were calculated by Eq. 1:

1 $$E_{{{\text{vs}} . {\text{RHE}}}} = E_{{{\text{vs}} . {\text{Ag/AgCl}}}} + 0.197\,{\text{V}} + 0.0592\,{\text{V}} + {\text{pH}}$$

The 0.197 V is $V_{{{\text{Ag/AgCl vs}} . {\text{ NHE}}}}^{\theta }$ at 25 °C, and the pH value of CO_2_-saturated 0.5 M KHCO_3_ solution is around 7.2.

### Calculation of CO F.E

The Faradaic efficiency (F.E.) was calculated by Eq. 2:

2 $${\text{F}} . {\text{E}} .= \frac{{x \times n \times N_{\text{A}} \times 2e}}{{I \times {t \mathord{\left/ {\vphantom {t e}} \right. \kern-0pt} e}}} \times 100\%$$

where *x* represents the concentration of CO (GC data); *n* corresponds to the amount of collected gas (the volume of the collected gas *V*_0_ is 1.0 mL), calculated via *n* = *PV*_0_/*RT* (*T* = 299.15 K, *P* = 1.013 × 10^5^ Pa, and *R* = 8.314 Nm K^−1^); Avogadro constant *N*_A_ = 6.02 × 10^23^ mol^−1^; the number of transfer electron is 2*e*; *I* (mA) represents the total current when collecting the pending tested gas; time (*t*) to collect 1.0 mL of gas is 3 s (CO_2_ flow rate is 20 mL min^−1^); *e* = 1.602 × 10^−19^ C e^−1^.

### Calculation of CO Turnover Frequency

In order to compare the catalytic activities of SAs–M–N–C with different metal concentrations, the turnover frequency (TOF) in SAs–M–N–C-catalyzed CO_2_ER was calculated according to Eq. 3 [30]:

3 $$TOF({\text{h}}^{ - 1} ) = \frac{{{{I_{\text{CO}} } \mathord{\left/ {\vphantom {{I_{\text{CO}} } {nF}}} \right. \kern-0pt} {nF}}}}{{m_{\text{catalyst}} \times {m \mathord{\left/ {\vphantom {m M}} \right. \kern-0pt} M}}} \times 3600$$

where *I*_CO_ is the partial current for CO production, A; *n* is the transferred number of electron during CO_2_ER, which is 2 for CO production; *F* is the Faradaic constant, 96,485 C mol^−1^; *m*_catalysts_ is the mass of catalysts loaded on the working electrode, which is 1.0 mg in our system; *m* is the metal concentration in SAs–M–N–C; *M* is the corresponding atomic mass of central metal.

The TOF values were calculated at the potential where the SAs–M–N–C delivered their maximum CO_2_ER performance for CO production. The TOF values of SAs–Fe–N–C, SAs–Co–N–C, SAs–Ni–N–C, and SAs–Cu–N–C for CO production were calculated to be 26.7, 11.3, 114.9, and 14.9 h^−1^, respectively, suggesting that the catalytic activities of SAs–M–N–C followed the sequence of Ni > Fe > Cu > Co.

## Results and Discussion

**Fig. 1:**
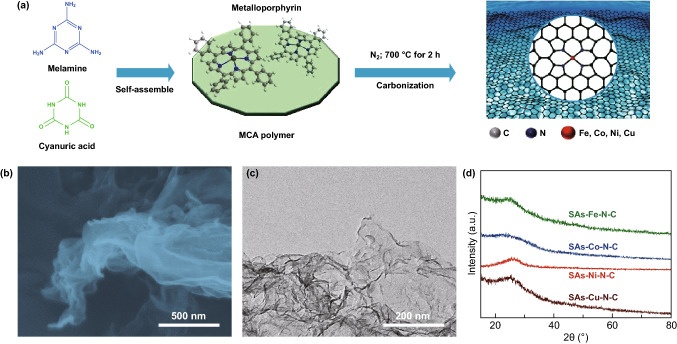
**a** Schematic diagram of synthetic process of SAs–M–N–C. **b**, **c** FESEM and TEM images of SAs–Ni–N–C. **d** XRD patterns of SAs–M–N–C

**Fig. 2:**
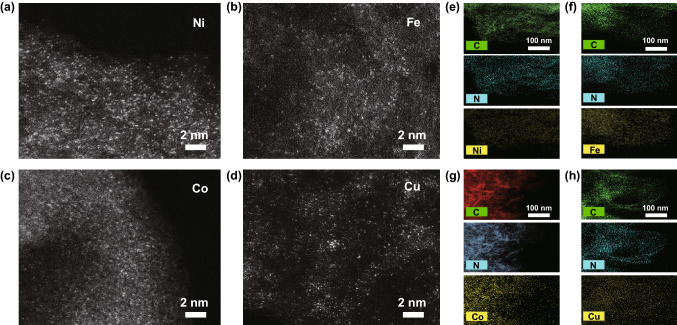
**a**–**d** AC HAADF-STEM images of SAs–Ni–N–C, SAs–Fe–N–C, SAs–Co–N–C, and SAs–Cu–N–C. **e**–**h** Elemental distribution mapping images from HAADF-HRTEM imaging of SAs–Ni–N–C, SAs–Fe–N–C, SAs–Co–N–C, and SAs–Cu–N–C

A family of SAs–M–N–C (M = Fe, Co, Ni, Cu) CO_2_ER catalysts were fabricated via a one-step in situ pyrolysis of metalloporphyrin molecules and MCA polymer derived from the polyreaction of M and CA. As shown in Fig. 1a, the M and CA precursors were firstly self-assembled to form MCA polymer, and then, the composite of metalloporphyrin loaded on MCA polymer was carbonized at 700 °C for 2 h under N_2_ atmosphere. During the carbonization process, the MCA polymer was gradually evolved into graphitized carbon nanosheets, while the local chemical environment of pyrrole-type M–N_4_ structures in metalloporphyrin molecules was well preserved and in situ anchored into the graphitic carbon frameworks. FESEM and TEM images of SAs–M–N–C showed ultrathin carbon nanosheets morphologies with a horizontal size of several hundred nanometers and a few-layer thickness (Figs. 1b–c and S1–S2), and no obvious metal nanoparticles (NPs) were formed on the nanosheets surface. The presence of typical D and G bands located at 1354 and 1582 cm^−1^ in Raman spectra confirmed the feature of graphitic carbon structure in SAs–M–N–C (Fig. S3). XRD patterns further excluded the existence of metal NPs in SAs–M–N–C with the absence of corresponding characteristic diffraction peaks of metallic phase (Fig. 1d). ICP-AES of SAs–M–N–C quantified the high loading amounts of central metals in the SAs–Fe–N–C, SAs–Co–N–C, SAs–Ni–N–C, and SAs–Cu–N–C with the corresponding values of 5.49, 3.69, 3.77, and 5.05 wt%, respectively (Table S1). Further, the AC HAADF-STEM images of SAs–M–N–C displayed the homogenously distributed and isolated bright dots with single atomic diameter of ~ 0.25 nm on the surface of carbon nanosheets, which could be attributed to the isolated metal atoms due to its larger atomic number than C or N atoms (Fig. 2a–d) [31–33]. Additionally, large-scale elemental distribution mapping images of SAs–M–N–C from the AC HAADF-STEM images displayed the uniform distribution of metal and N atoms on the carbon nanosheets, demonstrating the atomic dispersion of metal species and successful doping of N atoms into carbon frameworks (Fig. 2e–h), which was further supported by the emerged characteristic peaks of C, N, and metal elements in XPS survey spectra (Fig. S4). Based on the above results, it can be concluded that the isolated metal species in the SAs–M–N–C were atomically dispersed on the surface of graphitic carbon nanosheets with high density.

**Fig. 3:**
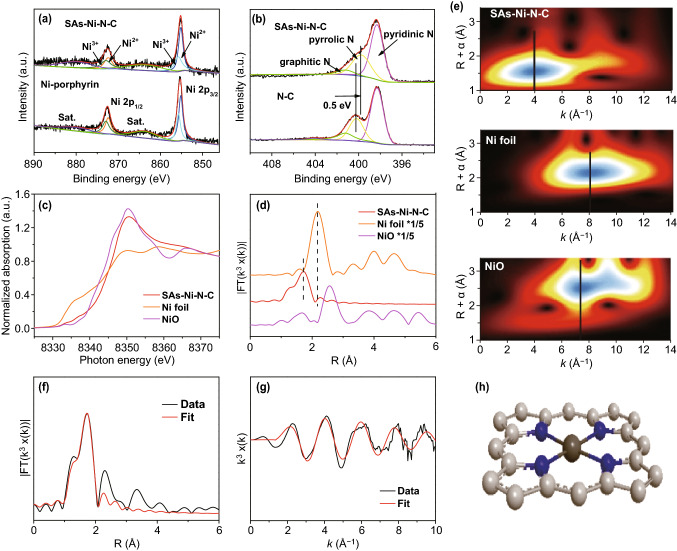
**a** High-resolution Ni 2*p* XPS spectra of SAs–Ni–N–C and Ni–porphyrin. **b** High-resolution N 1*s* XPS spectra of SAs–Ni–N–C and N–C. **c**–**e** Ni *k*-edge XANES spectra, Ni *k*-edge EXAFS spectra in R space, and WT-EXAFS images of SAs–Ni–N–C, Ni foil, and NiO. **f**, **g** FT-EXAFS fitting results of SAs–Ni–N–C. **h** Schematic diagram of local geometric structure in SAs–Ni–N–C

For the analysis of coordinated environment of metal atoms in SAs–M–N–C, taking the SAs–Ni–N–C as an example, high-resolution Ni 2*p*, N 1*s* XPS spectra, and XAS spectra were conducted. Figure 3a displays high-resolution Ni 2*p* XPS spectrum of SAs–Ni–N–C, in which the valence state of atomically dispersed Ni species was fitted to be Ni^2+^ and Ni^3+^ according to the peaks located at binding energies of 855.2/872.3 and 855.7/872.9 eV, respectively, whereas the Ni^0^ with the peak located at 852.6 eV was not observed [34], suggesting that the Ni species in SAs–Ni–N–C exist in an oxidized state instead of metallic Ni. This result was inconsistent well with XPS spectra of controlled Ni–porphyrin sample, in which the valence state of Ni species was also fitted to be Ni^2+^ and Ni^3+^ based on corresponding characteristic peaks, thus excluding the existence of metallic Ni^0^. Based on above results, it can be found that the valence state of Ni atoms was not transformed during the pyrolysis process, and the Ni species was well preserved in the atomic level and not aggregated into the Ni NPs. Additionally, high-resolution N 1*s* XPS spectra of SAs–Ni–N–C and pure N-doped carbon nanosheets (denoted as N–C, Fig. S5) that were synthesized under the same pyrolysis condition as SAs–Ni–N–C but free of adding Ni–porphyrin molecules are shown in Fig. 3b. As compared with N–C, the characteristic peak of pyrrolic N in SAs–Ni–N–C was chemically shifted with 0.5 eV, whereas no changes on the characteristic peaks of other types of N dopants were observed on SAs–Ni–N–C. These results demonstrated that the chemical environment of pyrrolic N in SAs–Ni–N–C was different from that in N–C sample, possibly originated from the formation of pyrrole-type Ni–N structures in SAs–Ni–N–C [35]. Such unique coordination category in SAs–Ni–N–C could be attributed to the reserved pyrrole-type Ni–N_4_ architectures originated from Ni–porphyrin molecules during pyrolysis, since high thermal stability of Ni–porphyrin preserved its local chemical structures [36], as confirmed by thermogravimetric analysis (TGA) results (Fig. S6). Further, the XAS spectra were used to accurately identify the local geometric structures of SAs–Ni–N–C at atomic level. Figure 3c displays the Ni *k*-edge X-ray absorption near edge structure (XANES) spectra of SAs–Ni–N–C with NiO and Ni foil as references, in which the adsorption edge energy of SAs–Ni–N–C was higher than that of Ni foil and NiO, indicating that the valence state of Ni species in SAs–Ni–N–C was a little bit higher than +2 [37, 38], inconsistent well with the fitting results of Ni^2+^ and Ni^3+^ from Ni 2*p* XPS spectra. The Fourier-transformed Ni *k*-edge *k*_3_-weighted extended X-ray absorption fine structure (EXAFS) in R space confirmed that the characteristic peak of Ni–Ni bonds from the Ni foil was located at 2.17 Å (Fig. 3d), which was absent in the spectrum of SAs–Ni–N–C, demonstrating the inexistence of Ni-based clusters/particles in the SAs–Ni–N–C. Meanwhile, the peaks ranged from 1.24 to 1.7 Å can be attributed to the first coordination shell of Ni–N bonds, which was inconsistent with the peaks in standard Ni–porphyrin [27, 30, 39, 40], suggesting the successful formation of Ni–N structures in the SAs–Ni–N–C. Since the characteristic peak of Ni–N bonds in Fig. 3d was located at the similar position with the Ni–O bonds in NiO, the wavelet transform (WT) of Ni *k*-edge EXAFS oscillations was further analyzed, and the results confirmed that the backscattering atoms bonded with Ni atom were indeed N atoms instead of O atoms because the WT-EXAFS analysis can provide the resolutions in both R and *k* spaces [21, 41]. In particular, the maximum intensity at 4.0 Å that associated with Ni–N bonds from SAs–Ni–N–C was quite different to that of Ni–O bonds at 7.5 Å in NiO (Fig. 3e). The fitting results of FT-EXAFS from SAs–Ni–N–C further revealed that the coordination number of Ni–N bonds was quantified to be four (Fig. 3f, g and Table S2). Based on the XPS and XAS fitting results, one can conclude that the local geometric structure of SAs–Ni–N–C was accurately confirmed to be the chemical configuration of single Ni atom coordinated with four pyrrolic N atoms (Fig. 3h). Likewise, the metal species in SAs–Fe–N–C, SAs–Co–N–C, and SAs–Cu–N–C were confirmed to be an oxidized state instead of metallic phase (Fig. S7); combined with the AC HAADF-STEM images of the above three catalysts, it can be deduced that the corresponding metal atoms were distributed with atomic level in SAs–Fe–N–C, SAs–Co–N–C, and SAs–Cu–N–C. Further, high-resolution N 1*s* spectra of SAs–Fe–N–C, SAs–Co–N–C, and SAs–Cu–N–C all exhibited a chemical shift of pyrrolic N characteristic peak with respect to that of the N–C (Fig. S8), demonstrating the formation of pyrrole-type M–N structures in the above three samples. Besides, the fitting results of FT-EXAFS from SAs–Fe–N–C, SAs–Co–N–C, and SAs–Cu–N–C all confirmed that the coordination number of M–N bonds in corresponding sample was quantified to be four (Figs. S9, S10 and Table S2), revealing that the local geometric structures in SAs–Fe–N–C, SAs–Co–N–C, and SAs–Cu–N–C were similar to that in SAs–Ni–N–C in the form of pyrrole-type M–N_4_ structures. These results demonstrated that this in situ pyrolysis of metalloporphyrin molecules loaded on surface of MCA polymer was a universal method to accurately synthesize atomically dispersed pyrrole-type M–N_4_ structures.

**Fig. 4:**
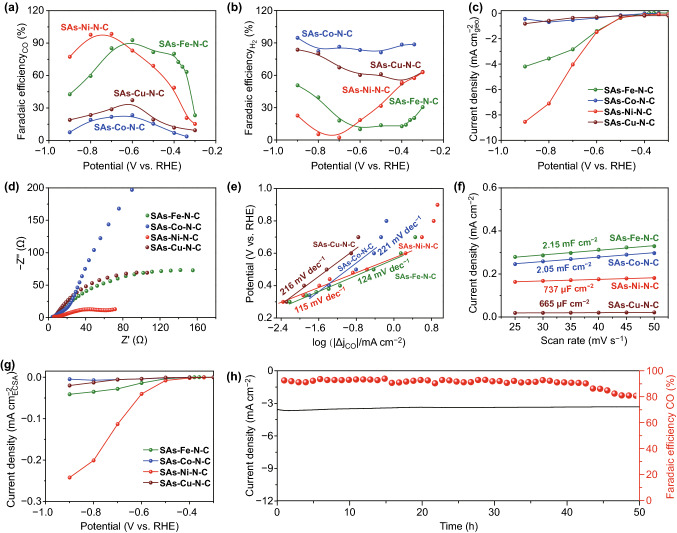
**a**–**g** F.E.s for CO and H_2_ productions, geometric surface area-normalized partial current density for CO production, Nyquist plot, Tafel slope, double-layer capacitance, and ECSA-normalized partial current density for CO production of SAs–Fe–N–C, SAs–Co–N–C, SAs–Ni–N–C, and SAs–Cu–N–C. **h** Long-term stability tests of 50 h, CO F.E., and current density from SAs–Ni–N–C-catalyzed CO_2_ER at − 0.7 V

The electrochemical CO_2_ER activity and selectivity of as-prepared SAs–M–N–C catalysts were performed in H-cell reactor with a typical three-electrode system. The linear sweep voltammetry (LSV) curves of SAs–M–N–C catalysts measured in CO_2_- and Ar-saturated 0.5 M KHCO_3_ solutions are shown in Fig. S11. Considering that the difference of delivered current densities between the CO_2_-saturated one and the Ar-saturated counterpart was originated from the CO_2_ER catalysis, the SAs–Ni–N–C displayed the highest catalytic activity for CO_2_ER among all investigated SAs–M–N–C samples. To further evaluate the selectivity of SAs–M–N–C for CO_2_ER by calculating Faradaic efficiency (F.E.), gaseous and liquid-phase products from CO_2_ER were quantified by gas chromatography (GC) and ^1^H nuclear magnetic resonance spectroscopy (^1^H NMR). Notably, the gaseous products produced from SAs–M–N–C-catalyzed CO_2_ER were identified to be CO and H_2_ gases, and the total F.E. of CO and H_2_ was calculated to be 100%, thus excluding the formation of liquid-phase products, as supported by ^1^H NMR results (Fig. S12). The corresponding F.E.s for CO and H_2_ products are shown in Fig. 4a, b, in which the SAs–Ni–N–C and SAs–Fe–N–C delivered a much higher CO F.E. than the SAs–Cu–N–C and SAs–Co–N–C under all the applied potentials, demonstrating high selectivity of SAs–Ni–N–C and SAs–Fe–N–C for CO_2_ER catalysis. Although the SAs–Ni–N–C exhibited a slightly lower CO_2_ER selectivity for CO generation than the SAs–Fe–N–C under the relatively positive potentials of − 0.3 ~ − 0.6 V, the former delivered a higher current density within this interval than the latter. Besides, with the applied potentials increased negatively, the CO F.E. of SAs–Ni–N–C was obviously superior to that of SAs–Fe–N–C and reached the maximum of 98.5% at − 0.7 V. The superior CO_2_ER performance of SAs–Ni–N–C with respect to SAs–Fe–N–C was further revealed by their partial current densities for CO production (Fig. 4c). These results suggested that, among the family of SAs–M–N–C catalysts, SAs–Ni–N–C was more suitable for the practical application in CO_2_ER because it delivered large current density and high selectivity during CO_2_ER process. From the results of electrochemical impedance spectroscopy (EIS) of SAs–M–N–C samples, the SAs–Ni–N–C possessed the lowest charge-transfer resistance among all the investigated catalysts for CO_2_ER, which was supported by the smallest radius in the Nyquist plot (Fig. 4d) [42]. Additionally, SAs–Ni–N–C-catalyzed CO_2_ER delivered a much lower Tafel slope of 115 mV dec^−1^ than that from the SAs–Fe–N–C (124 mV dec^−1^)-, SAs–Co–N–C (221 mV dec^−1^)-, and SAs–Cu–N–C (216 mV dec^−1^)-catalyzed counterparts (Fig. 4e), demonstrating the fastest reaction kinetics in SAs–Ni–N–C-catalyzed CO_2_ER process [22].

In order to clarify the intrinsic property of isolated M–N centers in SAs–M–N–C, the CO_2_ER performance of control N–C sample was evaluated (Fig. S13). Although the content of doped N species in N–C was much higher than that in SAs–M–N–C, the N–C exhibited a finite CO_2_ER performance in terms of catalytic activity, CO selectivity, and reaction kinetics with respect to SAs–M–N–C. This result indicated that the M–N centers played the key role in enhancing CO_2_ER performance. Furthermore, for precisely evaluating the intrinsic nature of SAs–M–N–C, electrochemical active surface area (ECSA) of SAs–M–N–C samples was calculated via measuring the corresponding double-layer capacitance (Figs. 4f and S14) [39, 43]. The ECSA of SAs–Fe–N–C, SAs–Co–N–C, SAs–Cu–N–C, and SAs–Ni–N–C was calculated to be 102, 98, 32, and 35 cm^2^, respectively. Despite the more exposed numbers of Fe–N and Co–N centers caused by the larger ECSAs in SAs–Fe–N–C and SAs–Co–N–C than that of Ni–N centers in SAs–Ni–N–C, the latter displayed the largest ECSA-normalized partial current density for CO production among the above investigated samples (Fig. 4g), suggesting that the superior CO_2_ER activity and selectivity of SAs–Ni–N–C are mainly attributed to high intrinsic property of the Ni–N sites, instead of other extrinsic factors, like ECSA. Besides, the ECSA-normalized partial current density for CO production by SAs–M–N–C samples also revealed the intrinsic property of M–N sites following the sequence of Ni–N > Fe–N > Cu–N > Co–N, inconsistent well with the sequence for CO selectivity. These results demonstrated that the CO_2_ER performance of SAs–M–N–C was strongly depend on the catalytic nature of central transition metal in M–N sites, giving the catalytic activity sequence of Ni > Fe > Cu > Co. Additionally, SAs–Ni–N–C also displayed a favorable CO_2_ER stability over 50 h of continuous reaction under a constant potential of -0.7 V (Fig. 4h), during which a slight attenuation was observed in both total geometric current density and CO F.E. after operated for 50 h, whereas the CO F.E. was still maintained at above 80%, illustrating a superior performance of SAs–Ni–N–C for catalyzing CO_2_ER with high selectivity and stability. Such excellent activity and stability of SAs–Ni–N–C for CO_2_ER catalysis were superior to that of the most previously reported M–N–C CO_2_ER electrocatalysts (Table S3).

**Fig. 5:**
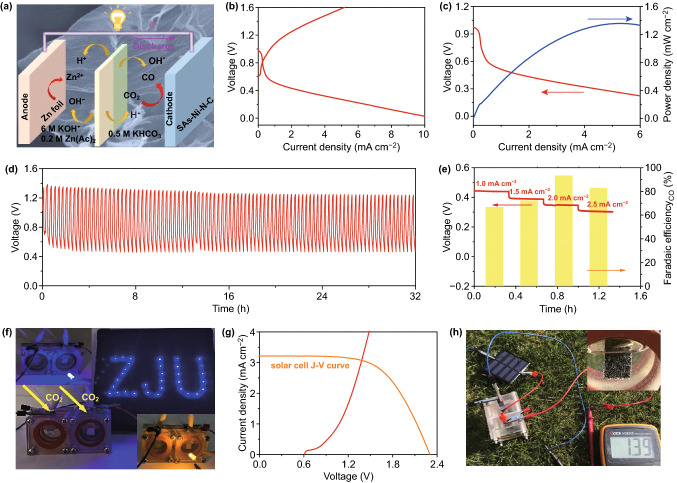
**a** Schematic diagram of the Zn–CO_2_ battery equipped with the SAs–Ni–N–C cathode. **b**, **c** Charge–discharge voltage curves and discharge and power density curves of the Zn–CO_2_ battery equipped with SAs–Ni–N–C cathode. **d** Charge–discharge cyclic curve of Zn–CO_2_ battery under constant current density of 1.0 mA cm^−2^. **e** CO F.E. of Zn–CO_2_ battery during galvanostatic discharge process. **f** Photograph of bulbs and homemade LED array lighted by two batteries in series. **g** Charge polarization curve and *J*–*V* curve of solar cell. **h** Photograph of Zn–CO_2_ battery charged by solar cell irradiated with natural sunlight

In order to further extend the practical application of CO_2_ER catalyzed by SAs–M–N–C, taking the SAs–Ni–N–C as an example, an aqueous Zn–CO_2_ battery composed of anode with Zn foil and cathode with SAs–Ni–N–C was designed to achieve the reaction of CO_2_ER along with the electricity output during its discharge process (Fig. 5a) [44–47], in which 6.0 M KOH with 0.2 M Zn(Ac)_2_ solution was used as anolyte and 0.5 M KHCO_3_ solution was used as catholyte; bipolar membranes were set to maintain the pH value of two chambers. The charge–discharge polarization curves shown in Fig. 5b demonstrated the rechargeable characterizations of this Zn–CO_2_ battery equipped with the cathode of SAs–Ni–N–C. Notably, the Zn–CO_2_ battery delivered a maximum power density of 1.4 mW cm^−2^ along with the current density of 5.3 mA cm^−2^ during the discharge process (Fig. 5c), which was much higher than the previously reported Zn–CO_2_ batteries equipped with the cathodes of atomically dispersed metal-anchored carbon materials (such as 0.21 mW cm^−2^ for NiPG [44] and 0.62 mW cm^−2^ for Cu–N_2_/GN nanosheets [47].) Meanwhile, the integrated Zn–CO_2_ battery with SAs–Ni–N–C cathode displayed a superior durability under cyclic charging and discharging processes with a constant current density of 1.0 mA cm^−2^ (Fig. 5d). Additionally, the CO_2_ER performance of SAs–Ni–N–C during discharge process of Zn–CO_2_ battery was identified, where the CO F.E. was achieved to the maximum of 93.3% under the power density of 0.7 mW cm^−2^ (Fig. 5e). Based on the above results, we conclude that the Zn–CO_2_ battery equipped with the SAs–Ni–N–C cathode can effectively realize the energy conversion of chemical energy into electric energy during its discharge process, that is, the occurring of the redox reactions achieved the reduction of CO_2_ into CO, and the electricity output was synchronously realized in the circuit. Consequently, both the bulbs and homemade LED array were lighted by the integrated Zn–CO_2_ battery (Fig. 5f). Furthermore, the emerged intersection at a voltage of 1.37 V between the charge polarization curves of this Zn–CO_2_ battery and the current–voltage (*J*–*V*) curve of solar cell (irradiated with Xe lamp, AM 1.5G, 100 mW cm^−2^) suggested the practical feasibility of using the solar cell to charge the Zn–CO_2_ battery (Fig. 5g). In this respect, solar cell irradiated under natural solar energy was used to effectually charge the Zn–CO_2_ battery, as featured by the occurring of anodic oxygen evolution reaction (Fig. 5h) [44, 46, 48], achieving the promising process of energy storage.

## Conclusions

In summary, we developed a universal principle for in situ pyrolysis of the MCA polymer-supported metalloporphyrin molecules to synthesize a family of atomically dispersed SAs–M–N–C catalysts. The experimental results revealed that the isolated metal species was bonded by pyrrolic N atoms and atomically distributed on the ultrathin carbon nanosheets with accurate pyrrole-type M–N_4_ structures. Owing to the specific nature of pyrrole-type M–N_4_ structure in SAs–M–N–C catalysts, the CO_2_ER performances catalyzed by SAs–M–N–C followed the sequence of Ni > Fe > Cu > Co, in which the SAs–Ni–N–C catalyst exhibited an excellent performance in CO_2_ER with the measured onset potential and Tafel slope of − 0.3 V and 115 mV dec^−1^, along with the detected maximum CO selectivity and long-term stability of 98.5% and 50 h at the optimized − 0.7 V, respectively. Such superior CO_2_ER performance achieved by SAs–Ni–N–C was outperforming almost all of the previously reported M–N–C electrocatalysts. Additionally, an integrated Zn–CO_2_ battery equipped the SAs–Ni–N–C cathode achieved the energy conversion and output with the maximum CO F.E. of 93.3% and peak power density of 1.4 mW cm^−2^. The developed strategy for synthesizing accurate pyrrole-type M–N_4_ sites in SAs–M–N–C catalysts as introduced in this work may give an alternative approach to construct the structurally controllable M–N_4_ centers supported on carbon materials for other promising electrochemical reactions, like hydrogen evolution reaction, nitrogen reduction reaction, and oxygen evolution reaction.

## Electronic supplementary material

Below is the link to the electronic supplementary material.

Supplementary material 1 (PDF 966 kb)
