# Correction to: A Universal Principle to Accurately Synthesize Atomically Dispersed Metal–N_4_ Sites for CO_2_ Electroreduction

**DOI:** 10.1007/s40820-020-00463-9

**Published:** 2020-06-24

**Authors:** Wanzhen Zheng, Feng Chen, Qi Zeng, Zhongjian Li, Bin Yang, Lecheng Lei, Qinghua Zhang, Feng He, Xi-Lin Wu, Yang Hou

**Affiliations:** 1 Key Laboratory of Biomass Chemical Engineering of Ministry of Education, College of Chemical and Biological Engineering, Zhejiang University, Hangzhou, 310027 People’s Republic of China; 2 College of Geography and Environmental Science, Zhejiang Normal University, Jinhua, 321004 People’s Republic of China; 3 College of Environment, Zhejiang University of Technology, Hangzhou, 310014 People’s Republic of China; 4 Institute of Zhejiang University - Quzhou, Quzhou, 324000 People’s Republic of China; 5 Ningbo Research Institute, Zhejiang University, Ningbo, 315100 People’s Republic of China; 6 Zhejiang Provincial Key Laboratory of Advanced Chemical Engineering Manufacture Technology, College of Chemical and Biological Engineering, Zhejiang University, Hangzhou, 310027 People’s Republic of China

## Correction to: Nano-Micro Lett. (2020) 12:108 10.1007/s40820-020-00443-z

In the original publication, the author name was incorrectly published as Xilin Wu. The correct author name should be Xi-Lin Wu, which is provided in this correction.

