# Supplementary material for: A Universal Principle to Accurately Synthesize Atomically Dispersed Metal–N4 Sites for CO2 Electroreduction
Source: Nanomicro Lett. 2020 May 9;12:108. doi: 10.1007/s40820-020-00443-z (PMC7770888; doi:10.1007/s40820-020-00443-z)
Supplement: Supplementary file 1 — Supplementary material 1 (PDF 966 kb) [file 40820_2020_443_MOESM1_ESM.pdf]

Supporting Information for

# A Universal Principle to Accurately Synthesize Atomically Dispersed Metal-$N_4$ Sites for $CO_2$ Electroreduction

Wanzhen Zheng[1, 3], Feng Chen[2], Qi Zeng[1], Zhongjian Li[1], Bin Yang[1, 4], Lecheng Lei[1, 4], Qinghua Zhang[6], Feng He[3], Xilin Wu[2, *], Yang Hou[1, 4, 5, *]

[1]Key Laboratory of Biomass Chemical Engineering of Ministry of Education, College of Chemical and Biological Engineering, Zhejiang University, Hangzhou 310027, People's Republic of China

[2]College of Geography and Environmental Science, Zhejiang Normal University, Jinhua 321004, People's Republic of China

[3]College of Environment, Zhejiang University of Technology, Hangzhou 310014, China

[4]Institute of Zhejiang University - Quzhou, Quzhou 324000, People's Republic of China

[5]Ningbo Research Institute, Zhejiang University, Ningbo 315100, People's Republic of China

[6]Zhejiang Provincial Key Laboratory of Advanced Chemical Engineering Manufacture Technology, College of Chemical and Biological Engineering, Zhejiang University, Hangzhou 310027, People's Republic of China

*Corresponding authors. E-mail: yhou@zju.edu.cn (Yang Hou); dbwxl@zjnu.cn (Xilin Wu)

## Supplementary Figures

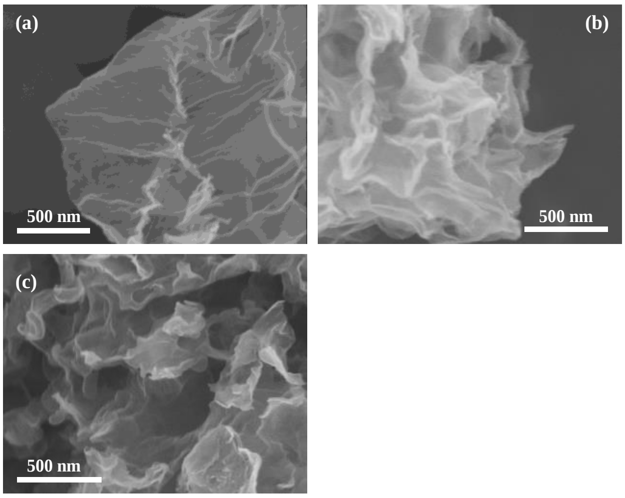


**Fig. S1** FESEM images of (**a**) SAs-Fe-N-C, (**b**) SAs-Co-N-C, (**c**) SAs-Cu-N-C

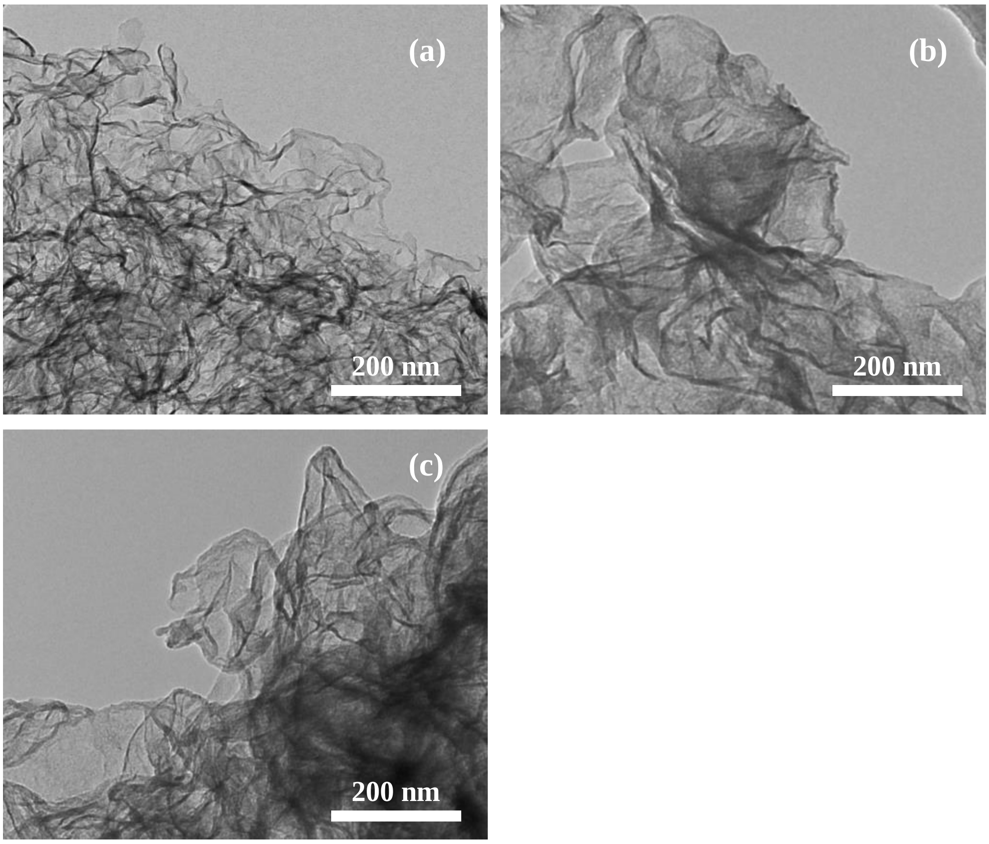


**Fig. S2** TEM images of (**a**) SAs-Fe-N-C, (**b**) SAs-Co-N-C, (**c**) SAs-Cu-N-C

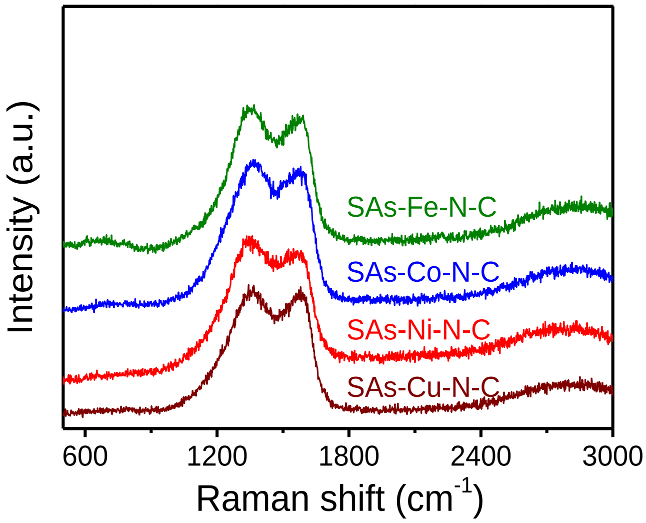


**Fig. S3** Raman spectra of SAs-Fe-N-C, SAs-Ni-N-C, SAs-Co-N-C, and SAs-Cu-N-C

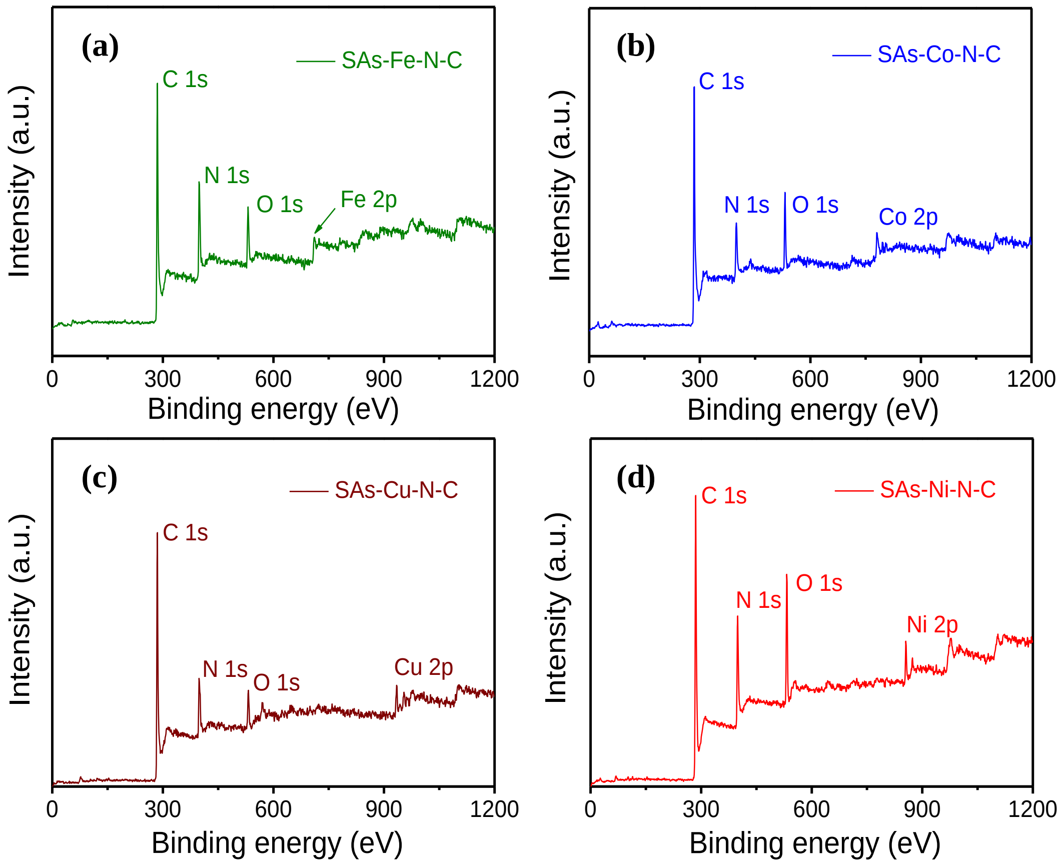


**Fig. S4** XPS survey spectra of (**a**) SAs-Fe-N-C, (**b**) SAs-Co-N-C, (**c**) SAs-Cu-N-C, and (**d**) SAs-Ni-N-C

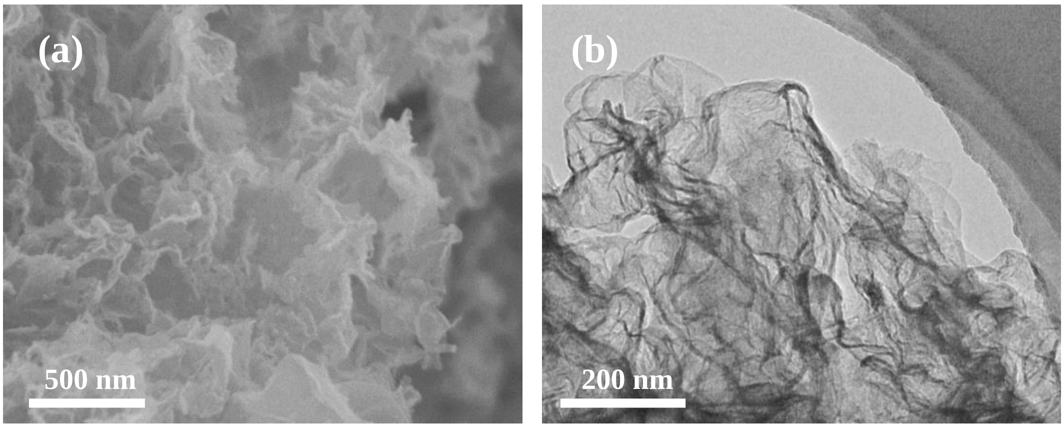


**Fig. S5** (**a**) FESEM and (**b**) TEM images of N-C

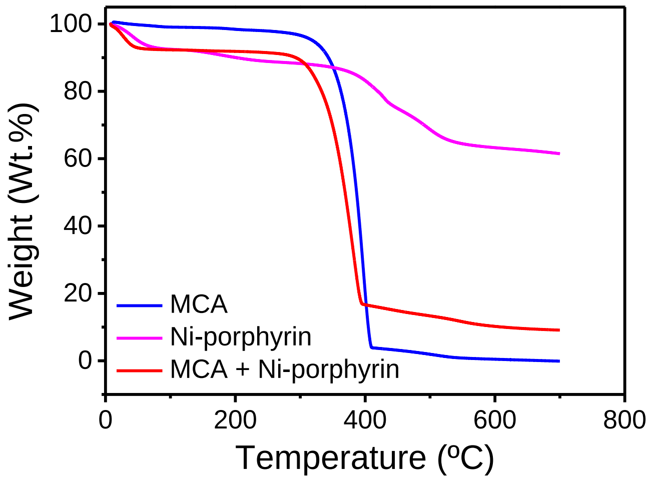


**Fig. S6** TGA curves of MCA, Ni-porphyrin, and composite of MCA and Ni-porphyrin

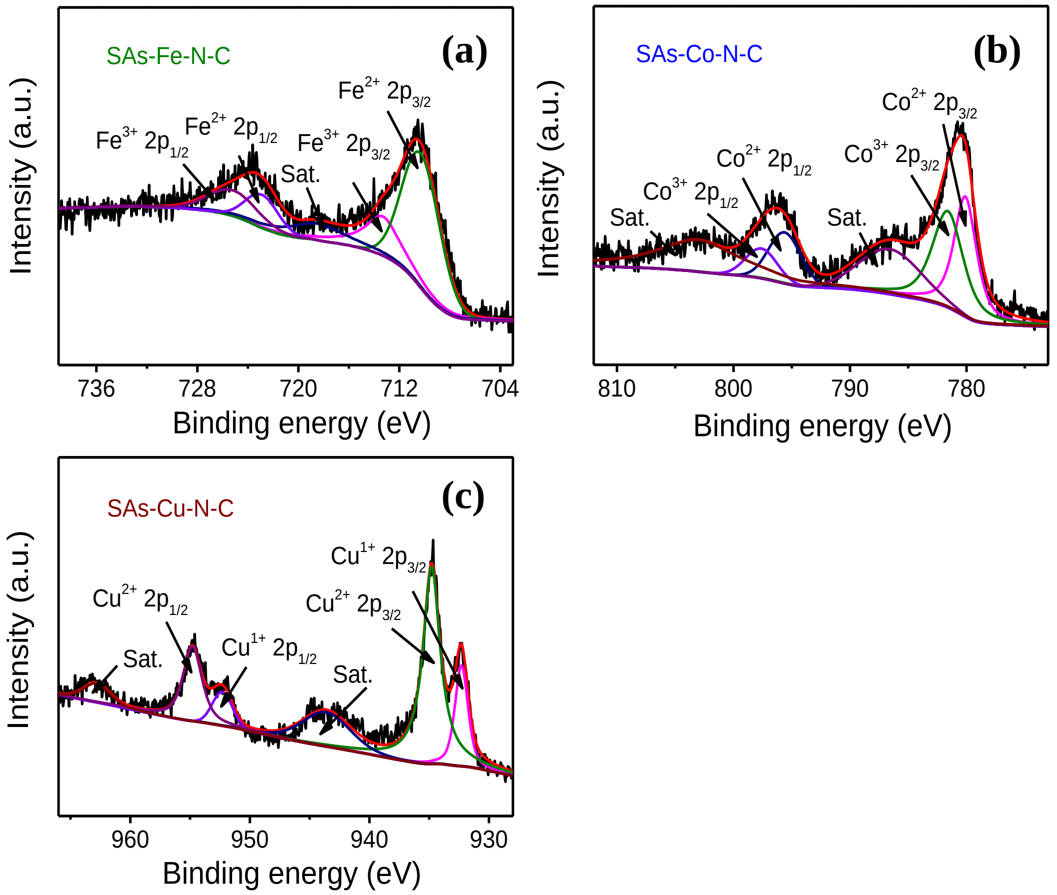


**Fig. S7** High resolution metal XPS spectra of (**a**) SAs-Fe-N-C, (**b**) SAs-Co-N-C, and (**c**) SAs-Cu-N-C

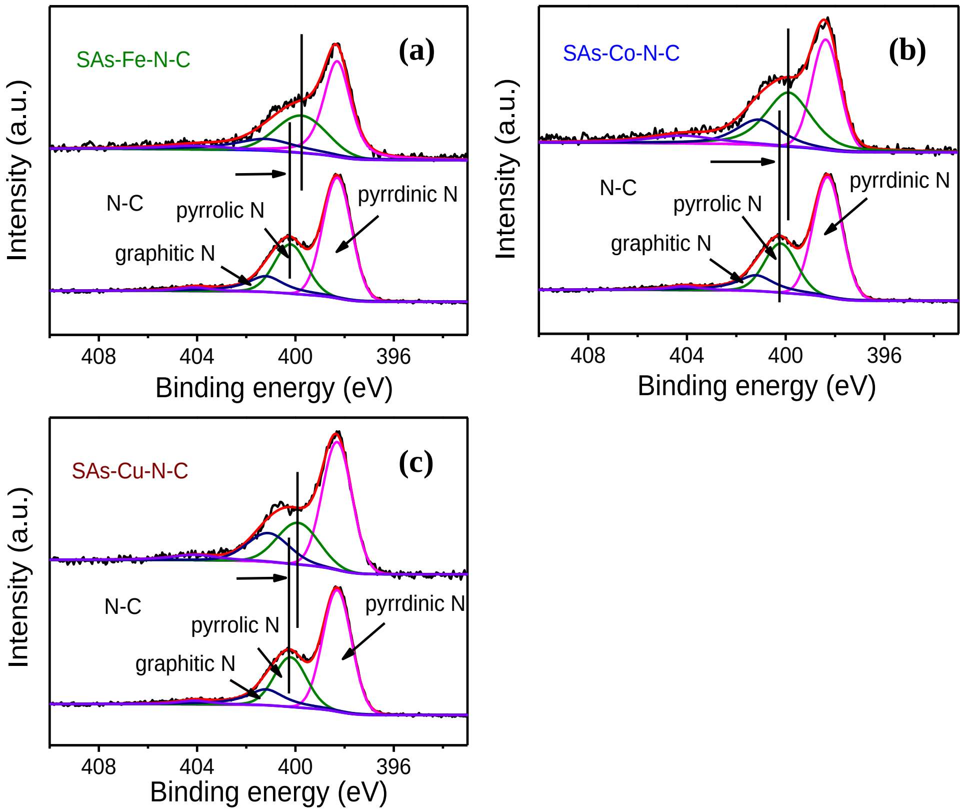


**Fig. S8** High resolution N 1s XPS spectra of (**a**) SAs-Fe-N-C, (**b**) SAs-Co-N-C, and (**c**) SAs-Cu-N-C

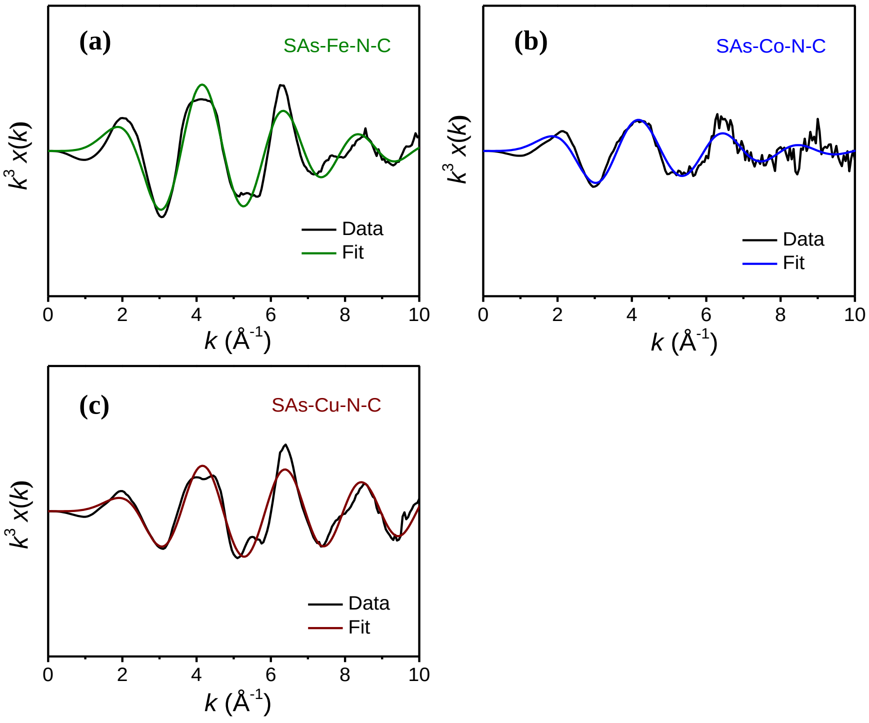


**Fig. S9** FT-EXAFS fitting results of (**a**) SAs-Fe-N-C, (**b**) SAs-Co-N-C, and (**c**) SAs-Cu-N-C

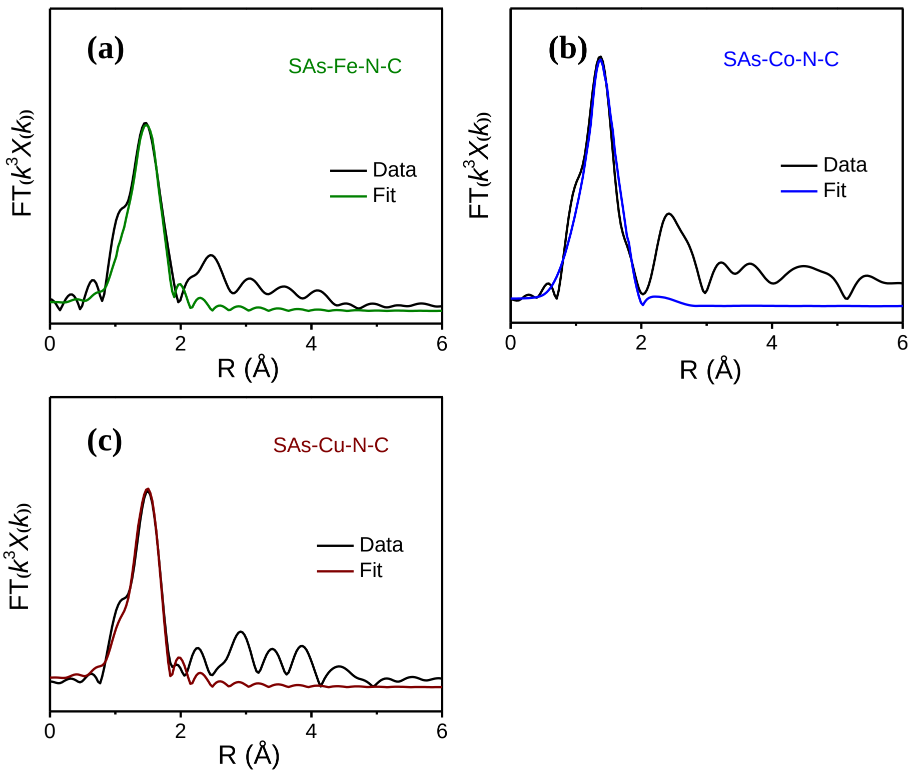


**Fig. S10** FT-EXAFS fitting results of (**a**) SAs-Fe-N-C, (**b**) SAs-Co-N-C, and (**c**) SAs-Cu-N-C

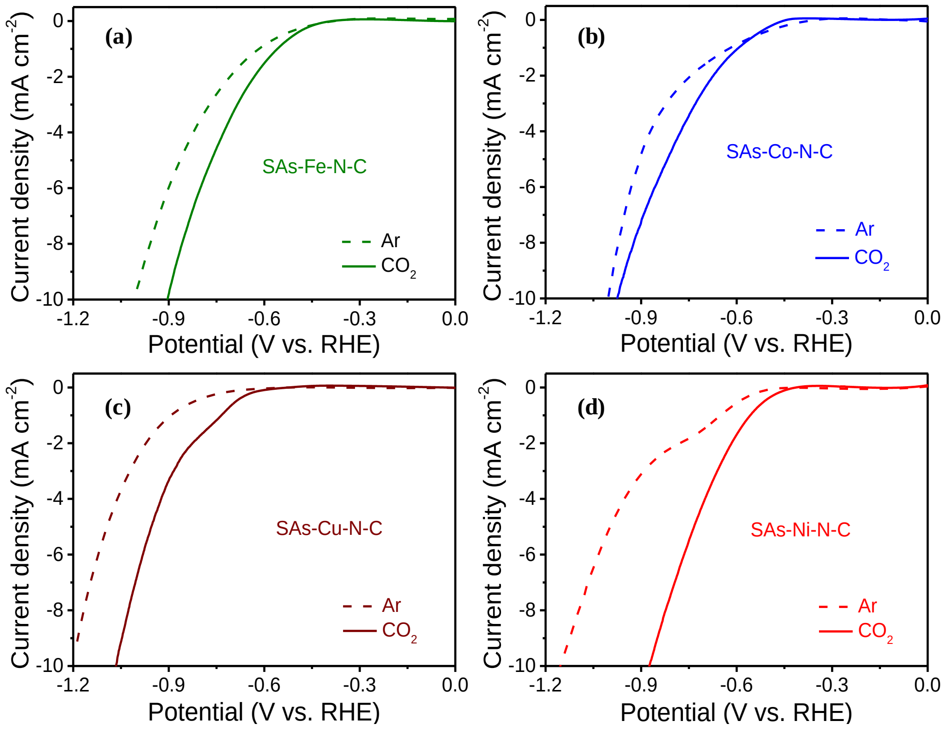


**Fig. S11** LSV curves measured in $CO_2$ and Ar saturated 0.5 M $KHCO_3$ solutions of (**a**) SAs-Fe-N-C, (**b**) SAs-Co-N-C, (**c**) SAs-Cu-N-C, and (**d**) SAs-Ni-N-C

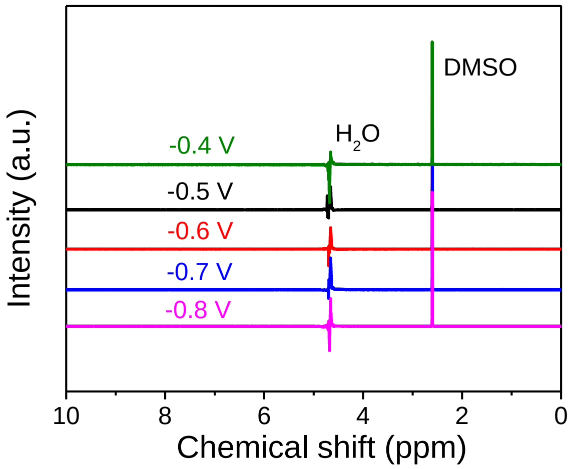


**Fig. S12** $^{1}$H NMR results of SAs-Ni-N-C catalyzed $CO_2$ER.

The total F.E. of gaseous CO and $H_2$ products in SAs-Ni-N-C catalyzed $CO_2$ER was calculated to be 100%. Likewise, the gaseous CO and $H_2$ products with 100% F.E. were observed for the SAs-Fe-N-C, SAs-Cu-N-C, and SAs-Co-N-C catalyzed one, respectively. Taking SAs-Ni-N-C as an example, all liquid-phase products under investigated potentials were measured by $^{1}$H NMR spectra, during which the 0.5 M $KHCO_3$ electrolyte after long-term $CO_2$ER electrolysis was mixed with internal standard of DMSO in $D_2O$, and then the mixture was directly identified on $^{1}$H NMR. As shown in Fig. S12, the $^{1}$H NMR spectra of 0.5 M $KHCO_3$ electrolyte from the SAs-Ni-N-C catalyzed $CO_2$ER under applied potentials only exhibited the signals of $H_2O$ and DMSO, no characteristic peaks of any liquid-phase $CO_2$ER products was observed, which excluded the formation of liquid-phase products in the SAs-Ni-N-C catalyzed $CO_2$ER electrolysis.

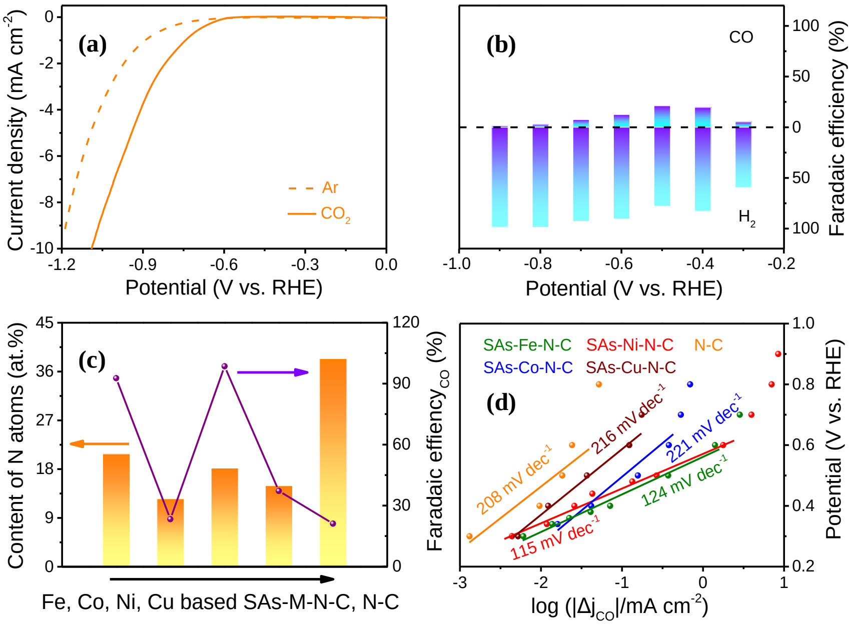


**Fig. S13** LSV curves measured in $CO_2$ and Ar saturated 0.5 M $KHCO_3$ solutions for (**a**) N-C, (**b**) F.E. of CO and $H_2$ products of N-C, (**c**) content of N dopants and

maximum CO F.E. and (**d**) Tafel slopes of SAs-Fe-N-C, SAs-Co-N-C, SAs-Ni-N-C, SAs-Cu-N-C, and N-C

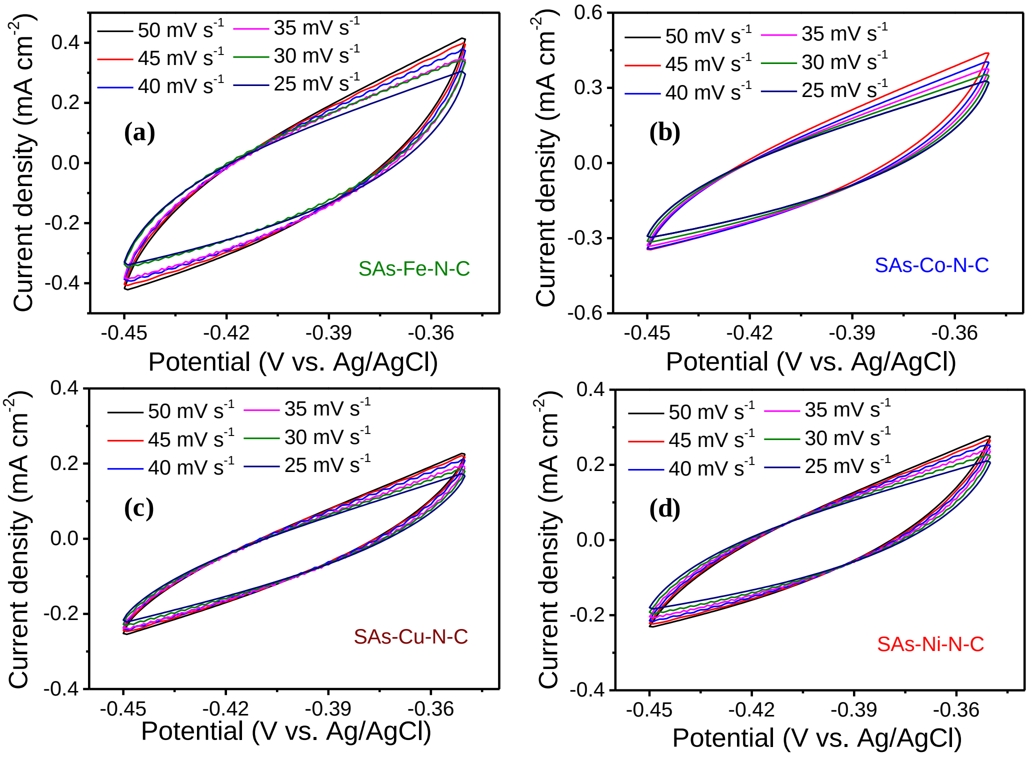


**Fig. S14** CV curves measured with different scan rates at the potential of -0.35 V ~ -0.45 V vs. Ag/AgCl of (**a**) SAs-Fe-N-C, (**b**) SAs-Co-N-C, (**c**) SAs-Cu-N-C, and (**d**) SAs-Ni-N-C

The ECSA was calculated by the following equation [S1]:

$$\mathrm{ECSA} = R_f \cdot S$$

The $R_f$ (roughness factor) is calculated by $R_f = C_{dl}/C_s$, where $C_s$ is the specific capacitance (21 μF $cm^{-2}$) of graphene [S2], S is the geometric active area (1 × 1 $cm^2$). The ECSA of SAs-Fe-N-C, SAs-Co-N-C, SAs-Cu-N-C, and SAs-Ni-N-C were calculated to be 102, 98, 32, and 35 $cm^2$, respectively.

**Table S1** Contents of center metal species in the SAs-M-N-C quantified by ICP-AES measurements

| Samples | Weight (W: g) | metered volume ($V_0$: mL) | Concentration (C: mg $L^{-1}$) | Dilution factor (I) | Content of metal (wt%) |
|---|---|---|---|---|---|
| SAs-Fe-N-C | 0.0079 | 10 | 2.17 | 20 | 5.49 |
| SAs-Co-N-C | 0.0217 | 10 | 1.60 | 50 | 3.69 |
| SAs-Ni-N-C | 0.0514 | 10 | 1.94 | 100 | 3.77 |
| SAs-Cu-N-C | 0.0208 | 10 | 2.10 | 50 | 5.05 |

*Calculation of the metal amount in the SAs-M-N-C:

Taking SAs-Ni-N-C as an example, the content of metal quantified by ICP-AES was calculated with the following formula:

$$Ni\ (wt\%) = \frac{C \times V_0 \times 10^{-6}}{W} \times I \times 100\% = \frac{1.94 \times 10 \times 10^{-6}}{0.0514} \times 100 \times 100\% = 3.77\ wt\%$$

**Table S2** Structural parameters of SAs-Ni-N-C, SAs-Fe-N-C, SAs-Co-N-C, and SAs-Cu-N-C extracted from the EXAFS fitting results. ($S_0^2 = 0.85$)

| Sample | Scattering pair | CN | R(Å) | $\sigma^2$ ($10^{-3}$Å$^2$) | $\Delta E_0$ (eV) | R factor |
|---|---|---|---|---|---|---|
| SAs-Ni-N-C | Ni-N | 4.1±0.6 | 2.10±0.02 | 4.6±0.9 | -3.5±1.1 | 0.01 |
| SAs-Fe-N-C | Fe-N | 4.1±0.6 | 1.91±0.02 | 4.7±0.9 | -3.5±1.1 | |
| SAs-Co-N-C | Co-N | 3.8±0.9 | 1.90±0.02 | 6.4±1.0 | -5.7±1.1 | |
| SAs-Cu-N-C | Cu-N | 4.1±0.7 | 1.92±0.02 | 5.6±0.6 | -4.1±0.8 | |

$S_0^2$ is the amplitude reduction factor; CN is the coordination number; R is interatomic distance (the bond length between central atoms and surrounding coordination atoms); $\sigma^2$ is Debye-Waller factor (a measure of thermal and static disorder in absorber-scatterer distances); $\Delta E_0$ is edge-energy shift (the difference between the zero kinetic energy value of the sample and that of the theoretical model). R factor is used to value the goodness of the fitting.

**Table S3** Comparison of $CO_2$ER performance and stability of SAs-Ni-N-C with other reported M-N-C catalysts for CO production

| Catalyst | Onset potential (V vs. RHE) | F.E. (%) | Tafel slope (mV dec$^{-1}$) | Stability (h) | Ref. |
|---|---|---|---|---|---|
| SAs-Ni-N-C | -0.3 | 98.5 | 115 | 50 | This work |
| $Fe^{3+}$-N-C | -0.2 | 95.0 | 117 | 12 | [S3] |
| Ni-$N_4$-C | -0.4 | 99.0 | 103 | 30 | [S4] |
| NiN-GS | -0.35 | 93.2 | 139 | 20 | [S5] |
| Ni SAs/N-C | -0.57 | 71.9 | 249 | 60 | [S6] |
| Ni-NG | -0.31 | 95.0 | 110 | 20 | [S7] |
| $Ni^{2+}$@NG | -0.38 | 92.0 | 142 | 20 | [S8] |
| Ni-NCB | -0.4 | 99.0 | 101 | 24 | [S9] |
| Co-TPP | -0.46 | 83.0 | 255 | 4 | [S10] |
| Cu–$N_2$/GN | -0.33 | 81.0 | 245 | 10 | [S11] |

## Supplementary References

[S1] S. Gao, Y. Lin, X. Jiao, Y. Sun, Q. Luo et al., Partially oxidized atomic cobalt layers for carbon dioxide electroreduction to liquid fuel. Nature **529**(7584), 68-71 (2016). https://doi.org/10.1038/nature16455

[S2] H.B. Yang, S.F. Hung, S. Liu, K. Yuan, S. Miao et al., Atomically dispersed Ni (I) as the active site for electrochemical $CO_2$ reduction. Nat. Energy **3**(2), 140-147 (2018). https://doi.org/10.1038/s41560-017-0078-8

[S3] J. Gu, C.S. Hsu, L. Bai, H. Chen, X. Hu, Atomically dispersed $Fe^{3+}$ sites catalyze efficient $CO_2$ electroreduction to CO. Science **364**(6445), 1091 (2019). https://doi.org/10.1126/science.aaw7515

[S4] X. Li, W. Bi, M. Chen, Y. Sun, H. Ju et al., Exclusive ni-n4 sites realize near-unity CO selectivity for electrochemical $CO_2$ reduction. J. Am. Chem. Soc. **139**(42), 14889-14892 (2017). https://doi.org/10.1021/jacs.7b09074

[S5] K. Jiang, S. Siahrostami, A.J. Akey, Y. Li, Z. Lu et al., Transition-metal single atoms in a graphene shell as active centers for highly efficient artificial photosynthesis. Chem **3**(6), 950-960 (2017).

https://doi.org/10.1016/j.chempr.2017.09.014

[S6] C. Zhao, X. Dai, T. Yao, W. Chen, X. Wang et al., Ionic exchange of metal-organic frameworks to access single nickel sites for efficient electroreduction of $CO_2$. J. Am. Chem. Soc. **139**(24), 8078-8081 (2017). https://doi.org/10.1021/jacs.7b02736

[S7] K. Jiang, S. Siahrostami, T. Zheng, Y. Hu, S. Hwang et al., Isolated Ni single atoms in graphene nanosheets for high-performance $CO_2$ reduction. Energy Environ. Science **11**(4), 893-903 (2018). https://doi.org/10.1039/c7ee03245e

[S8] W. Bi, X. Li, R. You, M. Chen, R. Yuan et al., Surface immobilization of transition metal ions on nitrogen-doped graphene realizing high-efficient and selective $CO_2$ reduction. Adv. Mater. **30**(18), e1706617 (2018). https://doi.org/10.1002/adma.201706617

[S9] T. Zheng, K. Jiang, N. Ta, Y. Hu, J. Zeng, J. Liu, H. Wang, Large-scale and highly selective $CO_2$ electrocatalytic reduction on nickel single-atom catalyst. Joule. **3**(1), 265-278 (2019). https://doi.org/10.1016/j.joule.2018.10.015

[S10] X Hu, M. Ronne, S.U. Pedersen, T. Skrydstrup, K. Daasbjerg. Enhanced catalytic activity of cobalt porphyrin in $CO_2$ electroreduction upon immobilization on carbon materials. Angew. Chem. Int. Ed. **56**(23), 6468-6472 (2017). https://doi.org/10.1002/anie.201701104

[S11] W. Zheng, J. Yang, H. Chen, Y. Hou, Q. Wang et al., Atomically defined undercoordinated active sites for highly efficient $CO_2$ electroreduction. Adv. Funct. Mater. **30**(4), 1907658 (2019). https://doi.org/10.1002/adfm.201907658
